# Can Behavior Analysis Help Us Understand and Reduce Racism? A review of the Current Literature

**DOI:** 10.1007/s40617-020-00411-4

**Published:** 2020-02-12

**Authors:** Kozue Matsuda, Yors Garcia, Robyn Catagnus, Julie Ackerlund Brandt

**Affiliations:** The Chicago School of Professional Psychology, 325 N. Wells St, Chicago, IL 60654 USA

**Keywords:** acceptance and commitment training, cultural diversity, prejudice, racism, relational frame theory

## Abstract

Despite ongoing efforts to eradicate racism, it persists globally, negatively affecting education, mental health, community relations, and economic development. Every behavior analyst can, and should, contribute to the reduction of racism in some way. Unfortunately, little behavior-analytic research exists to guide us. This article proposes ways that members of our scientific community can learn about racism from a behavioral perspective, extend experimental analyses of prejudice, and intervene to reduce racism in varied settings. It describes both traditional behavior-analytic and functional-contextualist accounts of racism and summarizes the small amount of related empirical and applied research. The review suggests that combining traditional behavior-analytic methods with acceptance and commitment training techniques may attenuate racism more effectively. The article ends with a call to collaborate around this globally important issue—and to do more to reduce racism.

The authors of this article are diverse across various domains of ethnicity, nationality, and gender. To some extent, we vary in our philosophical orientations toward behavior science. However, because each is interested in ameliorating racism and discrimination, we originally planned a literature review to explore the topic more deeply. We searched 30 years of literature across 14 behavior-analytic studies, using keywords related to racism (see the Appendix for search parameters). Here are the results: zero.

We then changed the focus of the review to include conceptual articles about racism or prejudice and added functional-contextual studies of racism. Still, the sparse findings highlight a notable deficit in behavior analysis regarding racism. Consequently, this article invites readers to reduce racism with behavior analysis—through any professional role and from all theoretical orientations. Toward this goal, we share information to help those interested in racism learn about terms and behavioral perspectives (Part I), extend experimental analyses of prejudice (Part II), apply promising racism reduction strategies (Part III) and discuss how behavior analysis call for collaboration around racism.

An understanding of racism could lead us to interventions that ameliorate its adverse, deeply personal effects. Racism inhibits economic development, hinders academic achievement, and causes mental health problems such as depression and anxiety (Cohen, Garcia, Apfel, & Master, 2006). Behavior analysis, rooted in radical behaviorism, seeks to understand the complexities of human behavior, including social issues such as racism and prejudice (Dixon, Belisle, Rehfeldt, & Root, 2018). Unfortunately, little behavior-analytic research has focused on the topics of racism and prejudice^1^ (Arhin & Thyer, 2004; Briggs & Paulson, 1996). Although some articles on race were published in the 1970s, few have been published since (see Hauserman, Walen, & Behling, 1973, for an example). Because so few studies have been conducted in fields related to behavior analysis, many questions remain unanswered. We hope to pique readers’ curiosity and inspire collaboration across disciplines to reduce racism together. Thus, we begin by orienting readers with definitions and two behavioral views of prejudice.

## Part I: Understanding Racism, Prejudice, and Bias—Two Perspectives

We use *racism*, *prejudice*, and *racial bias* throughout the article. Behavior science lacks technical definitions for these words. Moreover, the terms are used interchangeably in everyday vernacular language, and they can apply to observable and private behaviors. For this article, we use the following definitions.

*Racism* is “a belief that one’s own racial or ethnic group is superior, or that other such groups represent a threat to one’s cultural identity, racial integrity, or economic well-being” (Racism, 2019a). It is also “a belief that race is the primary determinant of human traits and capacities and that racial difference produces an inherent superiority of a particular race” (Racism, 2019b).

*Prejudice* is a “preconceived opinion not based on reason or experience,” including “unreasoned dislike, hostility, or antagonism towards, or discrimination against, a race, sex, or other class of people” (Prejudice, 2019). Racial prejudice is a specific form of racism involving socially inappropriate and discriminatory behaviors, including verbal behavior, directed at members of an ethnic group (Briggs & Paulson, 1996; Lai et al., 2016).

Biased behavior is not inherently negative, and every human behaves in biased ways. Problems arise when biased behaviors lead to adverse outcomes for self and others. *Bias*, in the context of racism, is often used as a synonym for *prejudice*. It is an “inclination or prejudice for or against one person or group, especially in a way considered to be unfair”. Humans can, of course, overtly behave in biased ways. However, many psychology researchers study “implicit cognitive bias.” It is often a mentalistic, explanatory construct. However, behavior analysts define *implicit bias* as “behavior that is influenced in an implicit manner by cues that function as an indicator of the social group to which others belong” (De Houwer, 2019, p. 835), and the behavior is “implicit” if it occurs quickly in nonconscious or unintentional ways. The current article references many studies about this behavioral phenomenon, so we further explain the term in a later section.

### A Behavior-Analytic View of Racial Prejudice

How do people around the globe learn to exhibit beliefs, attitudes, and behaviors based on skin color or ethnoracial background? As noted, behavior-analytic studies related to racism and prejudice are regrettably scarce (see Arhin & Thyer, 2004). Nevertheless, research in behavior analysis may help us understand how racial prejudice originates and grows. A behavior-analytic account of bias may include contingencies of reinforcement or punishment, motivating operations, stimulus control, and verbal processes such as self-talk or rumination (see Critchfield, Barnes-Holmes, & Dougher, 2018, for a review). Racism and prejudice may develop, generally, through three basic direct-contingency principles: respondent, operant, and observational learning. In these three cases, *direct contingencies* mean that all behavior is learned through direct experience with the contingencies of reinforcement and under similar topographical stimulus control. (For interesting and thorough reviews of the behavior-analytic account of racism, see Arhin & Thyer, 2004, and Briggs & Paulson, 1996).

Respondent learning occurs when a neutral stimulus (NS) is paired with an unconditioned stimulus (UCS) and becomes a conditioned stimulus (CS), which elicits a conditioned response (CR). For example, an NS (a Black person) is paired with an immediate presentation of a UCS (a frightening murder), which produces a an unconditioned response UCR (horror or fear). A subsequent incident involving a Black individual may trigger racist responses such as “Blacks are frightening,” even though that individual was not involved in a murder.

Operant learning of racial prejudice occurs through reinforcement or punishment contingencies (Arhin & Thyer, 2004). For example, a person who gains attention and peer approval after expressing racist views is more likely to continue expressing such views (Briggs & Paulson, 1996). Observational learning involves direct observation or modeling of others’ racist behaviors (Skinner, Olson, & Meltzoff, 2019). For example, children whose parents make racist remarks or engage in race-based attacks may develop, practice, and perpetuate racially discriminatory behaviors.

Stimulus generalization is another process that may explain aspects of racism. Consider a child who watches a television show presenting White characters as brave police officers and Black characters as criminals. Repeatedly, on different shows, the White police officers arrest Black individuals. After such repeated exposures, the child may think or say that all White persons are brave, whereas Black persons are criminals. In this example, the child acquires a racial bias through exposure to the contingencies of reinforcement, and it generalizes to people who look alike.

To summarize, direct-contingency processes, including conditioning and stimulus generalization, do help explain racism from the behavior-analytic perspective. Researchers in the field of relational frame theory (RFT) offer an alternative account to the traditional behavior-analytic description of racism and prejudice. They describe related behavioral phenomena through a process of derived relational responding (DRR; Critchfield et al., 2018) and have published more extensively about racial prejudice. Behavior-analytic audiences could find inspiration in their work, so we provide a short primer in this article.

### A Functional-Contextual View of Racial Prejudice

Functional contextualism, a philosophical approach rooted in pragmatism, has commonalities with radical behaviorism. Yet it varies in terms of the focus on specific scientific goals, specifically predicting and influencing psychological occurrences with precision, scope, and depth (see Gifford & Hayes, 1999, for details). Stated differently, the accuracy of scientific analysis is defined by the effectiveness of prediction and influence (“what works”) and by its unit of analysis: the whole organism interacting with its environmental context. In functional contextualism, any method, procedure, or technique can be used as long as it contributes to the prediction and influence of behavior in a particular context.

Researchers in RFT work from the functional-contextualistic philosophical tradition, but behavior analysts should be aware of racism research conducted through the RFT lens. RFT suggests respondent, operant, and observational accounts of prejudice may not be complete without considering DRR processes (Critchfield et al., 2018; de Carvalho & de Rose, 2014; Dixon, Branon, Nastally, & Mui, 2009; McGlinchey & Keenan, 1997). *Derived* means acquiring some behaviors without direct exposure to contingencies of reinforcement. *Relational* means responding to one stimulus in terms of another stimulus—for instance, saying “faster” when asked to choose between options. Furthermore, humans learn derived stimulus relations. Take, for example, a child who learns that the contextual cue “brighter” means that something is more brilliant than another thing. After multiple exposures using the same contextual cue, a child may derive that light colors are “brighter” than “darker” colors. After further exposure, the same child relates that “brighter” means someone is smart or intelligent. Consequently, the relations become independent from the topographical character of the stimulus. In other words, arbitrary relations develop (Stewart, McElwee, & Ming, 2013).

Coordination or equivalence relations are the most basic form of DRR that may aid in the understanding of racial prejudice. For example, imagine a child learns an arbitrary relation between Stimulus A (Santa Claus) and Stimulus B (White person). Then, she sees a television show that portrays Stimulus C (being gentle and kind) with Stimulus A (Santa Claus). The child might then say, “White people are nice and kind,” “Santa Claus is White,” or “Kind people are like Santa Claus.” Other more advanced relations are equally likely—for example, opposition or comparison. In the previous example, the child may also learn that “unkind” is the opposite of “gentle.” Without further training, she might say, “I do not like people who are unkind,” or she may compare being gentle with other qualities—for instance, “Gentle people are nicer than unkind people.” Once a child is exposed to multiple social conventions, myriad relations are likely to emerge without additional exposure to the contingencies of reinforcement (Critchfield et al., 2018).

Research into derived stimulus relations may explain some complicated human social phenomena (Kohlenberg, Hayes, & Hayes, 1991; McGlinchey & Keenan, 1997; Roche, Barnes-Holmes, Barnes-Holmes, Stewart, & O’Hora, 2002). RFT describes multiple ways of relating stimulus events (Critchfield et al., 2018; Stewart et al., 2013) involved in the development of prejudice. From the perspective of RFT, racial prejudice involves a derived transformation of stimulus function across relations, based on direct or verbal contact with the functions of a few members of a specific group. For example, in the 1970s, people were introduced to Asian refugees fleeing by boat via frequent news reports referring to them as poor “boat people.” Those who have a history with these news reports may perceive Asians as poor, hardworking, or lacking in English proficiency. Conversely, people who frequently see Asian names on lists of successful businesspersons may see them as wealthy, cold, or intelligent. With these types of relations, RFT has been used to model racism and prejudice.

## Part II: Research About Racial Prejudice

Many functional-contextual studies have examined racism-related behaviors over the past decades (Barnes-Holmes, Murphy, Barnes-Holmes, & Stewart, 2010; Dixon et al., 2009; Watt, Keenan, Barnes, & Cairns, 1991). Stimulus equivalence and RFT research might explain how people acquire racist beliefs (Watt et al., 1991) or attitudes toward the color of skin (de Carvalho & de Rose, 2014; Mizael, de Almeida, Silveira, & de Rose, 2016) through the relations of social categorization (McGlinchey & Keenan, 1997).

Stimulus equivalence studies teach us that bias exists and can be measured behaviorally and that established biases are difficult to overcome, but we can explore ways to reduce bias experimentally in the laboratory. Several studies have demonstrated these findings, and we summarize a few in the following sections. We discuss the measurement of biased responding, the existence and persistence of bias, and examples of laboratory procedures to reduce bias.

### Measuring Bias

We need to find aspects of racial prejudice that we can measure in the laboratory if we hope to research it. Overt prejudiced behaviors, such as comments or actions that demonstrate bias, are observable (Guerin, 2003, 2005). In applied settings, many observable behaviors related to organizational, educational, and societal interactions could be measured as they relate to prejudicial or racist actions. In a rare empirical behavior-analytic study, educator bias was measured (Knochel, Blair, Kincaid, & Randazzo, 2019). Data collected on the rate of behavior-specific praise and reprimands to children of different demographic categories (White, Black, Latinx, Other) revealed prejudicial teacher behaviors. White students received more praise from teachers, whereas Black students received more reprimands from teachers.

Covert or implicit racial prejudice is more challenging to measure accurately—especially when participants may be socially motivated to appear less prejudiced. Researchers in social science and behavior analysis developed the implicit relational assessment procedure (IRAP) to measure implicit bias (Barnes-Holmes et al., 2010). The IRAP is a computer-based procedure for measuring implicit cognition, or the “relatively quick reactions that cohere with the respondent’s prevailing learning history in respect to the trial content” (Drake et al., 2015, p. 71). During each trial of a computerized IRAP, participants quickly respond to pairs of stimuli, such as words, pictures, or statements, in relation to contextual cues at the top of a computer screen. For example, one stimulus, an image or phrase at the top of the screen, has one relational cue right below it. Two response options are presented further down the screen, such as “correct” or “incorrect.” The participant selects one or the other.

The assessment often uses a relational elaboration and coherence model to assess a brief, immediate relational response (BIRR). That is, participants may display an instant response (i.e., a BIRR) under specific contextual (stimulus) control, which is said to reflect an implicit bias (Barnes-Holmes et al., 2010). In the IRAP, participants might respond to phrases such as “I think Black people are . . .” or “I think White people are . . .” by selecting responses that range from negative (e.g., stupid) to positive (e.g., intelligent; Power, Harte, Barnes-Holmes, & Barnes-Holmes, 2017). The test does not measure the relative strength, probability, or persistence of relational responses. Instead, response latency is measured by how many milliseconds elapse between the onset of the trial and the participant selecting a response (Barnes-Holmes et al., 2010; Kishita, Muto, Ohtsuki, & Barnes-Holmes, 2014). Slightly slower responding indicates stimulus pairings are not representative of a learning history or are inconsistent with a social norm for the respondent. Such responses are considered evidence of biased responding (Drake et al., 2015). In other words, if the participant’s response on a given trial is consistent with his or her BIRR, then his or her response should be quicker than when he or she must respond in an inconsistent manner.

Thus, we might use the IRAP to measure “how behavior (i.e., response times) can be influenced by race-related cues even when people do not have the intention to be influenced by those cues” (De Houwer, 2019, p. 838). In other words, bias is behavior. Despite theoretical differences about what the IRAP is said to measure, the test has emerged as one reliable and valid measure of relational repertoires as they relate to racial prejudice (Drake et al., 2015; Golijani-Moghaddam, Hart, & Dawson, 2013).

### DRR Research on the Existence and Persistence of Bias

Studies with the IRAP reveal something about how learning histories affect biased responding. Our learning histories create bias and make bias hard to overcome. Behavioral researchers have studied preexperimental learning histories to evaluate how much prior social learning interferes with forming new stimulus equivalence classes (Kohlenberg et al., 1991; McGlinchey & Keenan, 1997; Watt et al., 1991). The following examples illustrate learning history effects related to bias about ethnonationalist names or symbols and religion, race, or gender categorization. Overall, they provide evidence that racial biases exist and that changing them is difficult.

In one study, Northern Irish participants were trained to match three traditionally Northern Irish Catholic names to three 3-letter nonsense syllables and subsequently to match these nonsense syllables to three traditionally Protestant symbols. Sixty percent of those who had experienced ethnonationalist conflict failed to demonstrate expected laboratory-induced equivalence responses when Catholic names and Protestant symbols were conditionally related to common nonsense syllables (Watt et al., 1991). These results suggest that the preexperimental history of participants may have interfered with the development of equivalence classes when using socially loaded stimuli.

Another study examined the ability of younger participants with biases related to skin color to create equivalence classes with “positive” symbols and human faces of color. They pretested participants by asking them to match faces of Black and White men with either positive or negative symbols. They then trained participants to match positive and negative symbols to two geometric shapes, which were matched to Black faces and an arbitrary picture, respectively. Only one of four children exhibited an emergent relation between Black faces and positive symbols (de Carvalho & de Rose, 2014). The outcomes show that previous learning histories related to racism can be extremely challenging to overcome.

Another study in Northern Ireland examined social categorization bias among children and adults. Researchers trained Catholic and Protestant participants, ranging in age from primary school children to adults, to match a Protestant or Catholic stimulus to an arbitrary stimulus and then to match the arbitrary stimulus to a different Protestant or Catholic stimulus. They trained the stimulus classes in such a way that emergent relations would include both a Protestant and a Catholic member. None of the older participants demonstrated equivalence on the tests. However, the younger participants, who had shorter learning histories in their verbal communities, demonstrated equivalence (McGlinchey & Keenan, 1997).

In two other experiments, researchers trained participants to form stimulus classes that consisted of male and female names to create a transfer of contextual control across verbal relations that previously existed in the individuals’ repertoire and among experimentally derived equivalence classes. They found that the transfer of contextual control among stimulus classes was possible without specific training in these relations, both among previously learned verbal classes and among those that formed experimentally. The outcome suggests that decreasing social stereotyping requires changes in the contextual control of verbal relationships (Kohlenberg et al., 1991). Together, these studies tell us that learned biases are difficult to overcome in the laboratory, but it may be possible.

**Summary of DRR research about bias.** Basic research about DRR supports the idea that people learn to engage in racially prejudiced behaviors and that researchers can measure prejudice. It helps us understand bias as a process under specific contextual control. Researchers recreating and testing bias in laboratory settings help us understand how racial prejudice develops or persists. Interested researchers should extend these studies to decrease racial biases—and test alternative ones with new populations and methods. Racial prejudice is resistant to change, but some promising procedures have been developed.

### Research to Reduce Bias

If changing stereotypes or biases requires changing contextual control or verbal relationships, how do we do that? Several DRR studies demonstrate that stimulus equivalence training procedures may reduce bias, as measured by laboratory tests. For example, it is possible to change contextual features that control preestablished relations (Dixon & Lemke, 2007; Mizael et al., 2016) or expose people to counterstereotypical exemplars (Lai et al., 2016). We are not claiming that these procedures are changing prejudices in the long term, nor that they prevent overt racist behaviors outside the laboratory, but they may inform our thinking on the topic of potential interventions that can.

Several groups of researchers have investigated prejudice against Middle Eastern people. The first attempted to reduce prejudice with match-to-sample procedures (Dixon, Zlomke, & Rehfeldt, 2006b). Using a reinforcement procedure, researchers tried to train college students of American heritage to form relations between American and terrorist stimuli. The participants did not demonstrate the expected derived relations. Next, the researchers trained participants to match terrorist or American stimuli with “unity,” “peace,” and “resolve.” Under these conditions, participants were able to demonstrate some aspects of equivalence relations, though they still made frequent matching errors.

Two other studies demonstrated similar findings. First, U.S. citizens were trained to match Middle Eastern words, terrorist images, and American images (Dixon, Rehfeldt, Zlomke, & Robinson, 2006a, Dixon, Zlomke, & Rehfeldt, 2006b). In this study, too, the participants still made matching errors by responding in socially normative ways—for example, matching terrorist stimuli to terrorist stimuli—and derived relations were not observed. In another study, U.S. citizens were trained to match terrorist, American/terrorist mixed, and neutral flower images (Dixon, Rehfeldt, Zlomke, & Robinson, 2006a). The results suggest that participants had difficulty forming equivalence classes consisting of terrorist images and American images, yet they had no difficulty forming such classes involving only terrorist images. This study provides more evidence that preexisting relations or rules make forming new, contradictory relations difficult.

Another study suggests it may be possible to change racial prejudice about Middle Eastern people through these kinds of interventions, although learning histories make it hard (Dixon & Lemke, 2007). Researchers asked U.S. college students to rate Middle Eastern males, American males, and everyday objects using a Likert-type scale ranging from 1 (*evil*) to 10 (*good*). Participants demonstrated a prejudiced rating toward images of Middle Eastern males as *evil* during pretesting. However, after match-to-sample training, images of Middle Eastern males were rated closer to *good*, demonstrating that relational training can affect preexisting prejudice functions.

A more recent study investigated whether learning a new matching task might mitigate racial biases among children. The participants had shown “a pronounced negative bias toward Black faces before training” (Mizael et al., 2016, p. 451). The matching task was designed to create new equivalence relations between positive symbols and Black faces. After the procedure, all the children demonstrated the expected equivalence relations that were trained. More importantly, the learning process reduced the children’s negative biases toward Black faces (Mizael et al., 2016). The transfer of stimulus function may have reduced racial bias regarding skin color. Could interventions developed from these types of laboratory procedures be applied to reduce racism on a larger scale?

## Part III: Interventions to Reduce Racial Prejudice

The aforementioned studies described DRR laboratory protocols designed to reduce bias. What the research tells us is that adding more relations to the network, for example, “Latin people come to the United States to work, not to steal,” does not modify the preestablished biases. Instead, it makes them harder to extinguish. Maybe a different approach is needed, one that decreases the impact of rules and the transformation of function across the relational networks. Basic research on mindfulness procedures (Edwards et al., 2017; Hooper, Villatte, Neofotistou, & McHugh, 2010; Lueke & Gibson, 2015, 2016) has shown promising results in diminishing the dominance of verbal rules over problem behaviors. A relatively new procedure rooted in RFT principles that combines mindfulness and behavioral-based procedures called acceptance and commitment therapy (ACT) stems from the early 1980s (see Zettle, 2005, for a historical and empirical review). In the past few years, researchers have published studies on acceptance and commitment training (ACTr) for parent training (Corti et al., 2018; Gould, Tarbox, & Coyne, 2018; Hahs, Dixon, & Paliliunas, 2018; Pennefather, Hieneman, Raulston, & Caraway, 2018), gambling (Dixon, Wilson, & Habib, 2016), decreasing impulsivity (Dixon et al., 2019), reducing inflexible behavior (Szabo, 2019), teaching job interview skills (Brazeau et al., 2017), training staff (Castro, Rehfeldt, & Root, 2016; Chancey et al., 2018), and increasing academic performance (Paliliunas, Belisle, & Dixon, 2018). ACT may also help us intervene on critical social issues related to racial bias (Levin et al., 2016; Lillis & Hayes, 2007).

### What Is ACTr?

ACTr stands for the use of ACT procedures in diverse contexts other than traditional clinical settings. Few studies have assessed interventions to reduce bias or to attenuate the effects of racial bias on individuals. Most of those published focus on interventions with ACTr, which includes procedures and interventions that deal with private behaviors (Hoffmann, Contreras, Clay, & Twohig, 2016). The overall approach teaches people mindfulness, acceptance, and defusion strategies in the service of observable behaviors that align with their values, as well as skills for goal achievement.

ACTr interventions target six processes: the present moment, acceptance, defusion, self-as-context, values, and committed action. These are processes that help people be mindful and accepting of the present moment while remaining committed to changing their behavior to align with personal values (Hayes, Levin, Plumb-Vilardaga, Villatte, & Pistorello, 2013).

Contacting the *present moment* is fully focusing attention on our current experiences right now, rather than in the future or the past (Hoffmann et al., 2016). Research has shown that being present in the moment can decrease stress levels for caregivers of individuals with autism (Donnchadha, 2018), increase sustained attention for children (Enoch & Dixon, 2017), and reduce implicit racial bias (Lueke & Gibson, 2015). For example, individuals with autism better tolerated turn taking when taught to contact the present moment while waiting for their turn in a game. They learned to be present in the moment by paying attention to sounds, colors, and textures while waiting (Szabo, 2019).

*Acceptance* refers to actively engaging with the direct and derived effects of aversive functions as they occur (Hoffmann et al., 2016). For example, parents may face multiple aversive consequences when dealing with their children’s behavior. When implementing an extinction procedure, a parent might practice covert self-talk such as “I am hurting my child” or “I cannot handle this crying.” Through different metaphors and exercises, parents may be taught to engage with the aversive consequence of implementing behavioral procedures and accept them with less distress. Acceptance teaches us to feel or experience the current moment, as we engage with the present, without being overwhelmed by it or trying to avoid it. Studies suggest that acceptance-based procedures help parents cope with painful or unpleasant emotions during parenting (Gould et al., 2018), improve social isolation in mothers of children who have autism (Lunsky, Fung, Lake, Steel, & Bryce, 2018), and decrease parental stress levels (Corti et al., 2018).

*Defusion* disrupts the context that controls ineffective and rigid functions of private responding and teaches individuals to respond to the direct properties of the environmental stimuli (Hoffmann et al., 2016). In other words, it teaches us to experience or observe private events, such as thoughts and verbal behavior, without becoming caught up in them. When added to exposure and response-prevention procedures, defusion helped to decrease ritualistic behaviors in children with autism (Eilers & Hayes, 2015). Defusion has also helped people with autism reduce anxiety during practice job interviews (Brazeau et al., 2017) and decrease rigidity (Szabo, 2019). The process of defusion, along with other ACT processes, has also been investigated as a process to reduce racism (Lillis & Hayes, 2007).

*Self-as-context* refers to deictic responding, or the time and location context from which a speaker is behaving (McHugh, Stewart, & Almada, 2019). It is about perspective taking regarding our behavior (Leeming & Hayes, 2016). In other words, we can observe ourselves and our experiences separate from thinking, remembering, planning, and other covert verbal behaviors. This ability aids in developing a sense of self and learning advanced skills such as perspective taking and empathy. Perspective taking and empathy may relate to both prejudices (Levin et al., 2016) and combatting automatic expressions of discrimination by facilitating favorable racial contact experiences (Todd, Bodenhausen, Richeson, & Galinsky, 2011).

The last two processes are values and committed action. *Values* are rules that alter the reinforcing properties of other stimuli; they are verbal motivating operations that modify the appetitive or punishing functions of other events (Hoffmann et al., 2016). In other words, values require us to decide how we want to live our lives and what we care about most. For example, a student may say, “I want to help decrease racism in my school.” This statement can become a rule that alters the reinforcing value of engaging in behaviors such as connecting with local organizations to help combat racism or informing others about ways to help ethnic minorities in the school. *Committed actions* are the specific responses or steps taken to live up to our values. For example, we can value equality, but valuing it is not enough. Our actions should align with our values. Several strategies common to behavior analysis might help individuals behave in ways that align with their values, such as behavioral skills training or goal setting.

### Specific Interventions

ACTr procedures have been shown to reduce prejudice across different domains, with varying populations and practices (Krafft, Ferrell, Levin, & Twohig, 2018). A few promising behavior-analytic interventions may also reduce racial bias or attenuate the effects of bias. We loosely organized these into categories of mindfulness and acceptance, perspective taking, clarifying values, and behavior-analytic and combined approaches.

#### Mindfulness and acceptance

Mindfulness-based interventions may be promising for attenuating racial prejudice. Mindfulness is emerging as an evidence-based approach in psychology and behavior analysis. These procedures have been shown to help with a variety of behaviors, such as binge eating and weight loss (Godfrey, Gallo, & Afari, 2015; Katterman, Kleinman, Hood, Nackers, & Corsica, 2014), aggressive or disruptive behavior (Fix & Fix, 2013; Klingbeil et al., 2017; Shonin, Van Gordon, Slade, & Griffiths, 2013), and treatment of psychosis or schizophrenia (Khoury, Lecomte, Gaudiano, & Paquin, 2013). Mindfulness is defined in ACTr as “a skill set that allows one to contact fully the current environmental and personal context with open and flexible awareness” (Leeming & Hayes, 2016, p. 159), and it involves acceptance, defusion, the present moment, and perspective taking of self. ACTr practitioners also consider being present and engaging in perspective taking of self as processes associated with behavior change when combined with values and committed action (Leeming & Hayes, 2016).

Mindfulness, in RFT, is defined “as the defused, accepting, open contact with the present moment and the private events it contains as a conscious human being experientially distinct from the content being noticed” (Fletcher & Hayes, 2005, p. 322). Behavior analysts are beginning to integrate mindfulness-based procedures with commonly used behavioral techniques in our discipline. In such studies, behavior analysts define mindfulness in terms related to specific covert and overt behaviors associated with “mindfulness” and “mindfulness training” (Wolfgang & Catagnus, 2019). We can also investigate related interventions and their outcomes with traditional behavior-analytic experimental designs. Indeed, behavior-analytic researchers have been implementing mindfulness interventions in single-subject designs over the past few years (Wolfgang & Catagnus, 2019). Although the racial prejudice studies described next are not behavior analytic, they may inspire researchers to implement behavior-analytic mindfulness-based procedures.

Undergraduates completed a mindful-attention awareness scale and then listened to either mindfulness or control audio recordings before completing an implicit association test (IAT). The IAT provided a measure of the strength of automatic associations derived from reaction speeds during two classification tasks related to race and age. Those who listened to the mindfulness recordings exhibited increased mindfulness and decreased implicit race and age bias (Lueke & Gibson, 2015). A follow-up study assessed the benefits of a 10-min mindfulness meditation audiotape on discriminatory behavior (Lueke & Gibson, 2016). Participants in the mindfulness condition showed less discrimination than those in the two control conditions. IRAP researchers have used similar procedures (e.g., Edwards et al., 2017; Hooper et al., 2010; Kishita et al., 2014; Ritzert, Forsyth, Berghoff, Barnes-Holmes, & Nicholson, 2015).

#### Training perspective taking

ACTr and behavior-analytic researchers have developed various protocols to teach perspective taking, though typically for children (see, e.g., McHugh et al., 2019; Peters & Thompson, 2018). A lack of perspective taking is one potential predictor of generalized prejudice (Levin et al., 2016), so teaching and motivating people to take the perspective of out-group members may be an effective strategy for reducing racial bias. Perspective-taking strategies were shown to attenuate racial prejudice in college students, as assessed by IAT response (Todd et al., 2011). Participants adopted the perspective of an African American individual unfairly treated at a department store. The participants vividly visualized what the person might be thinking or feeling in such a situation or wrote a short narrative about a typical day in the life of someone from a minority group. The perspective-taking activities helped attenuate the automaticity of racially biased behaviors. Taking the perspective of others helps us identify with them more automatically (Todd, Bodenhausen, & Galinsky, 2012), reduces stereotyping and in-group favoritism (Galinsky & Moskowitz, 2000), and attenuates automatic expressions of racial bias (Todd et al., 2011). Logically, different types of behavior-analytic perspective-taking protocols could also help to reduce bias or racism.

Under some conditions, perspective taking produces adverse effects, depending on context and individual characteristics (Parker, Atkins, & Axtell, 2008), but combining it with mindfulness may ameliorate such issues. For example, in a study investigating brief perspective-taking interventions, perspective taking alone made prejudicial views toward the elderly more negative. Adding a mindfulness component ameliorated this effect to some extent (Edwards et al., 2017). Behavioral researchers interested in attenuating racial bias could further investigate both mindfulness and perspective-taking interventions.

#### Clarifying values

In ACTr, value clarification corresponds to extended behavioral patterns that lead individuals to powerful yet delayed reinforcers. Value intervention is not a new procedure in psychology, as social psychologists have used it to boost social connection and cooperation while reducing the adverse effects of stereotype threat. For example, different value clarification exercises improved grades and decreased racial achievement gaps for low-performing African American students (Cohen et al., 2006). The students wrote a series of essays in which they affirmed values of importance to them, and their grade point averages were positively affected even 2 years after the intervention. In a pilot intervention in a college setting, a short ACT-based lesson, but not a traditional lecture, led to some changes in how students responded on a prejudice-related questionnaire—particularly around positive behavioral intentions (Lillis & Hayes, 2007). Although the number of ACT-based studies using value clarification is limited, it may be a potential area for intervention research (Levin et al., 2016).

#### Behavior-analytic and combined approaches

More traditional interventions related to racial behavior also have potential (see Guerin, 2003, 2005). Consider the study described previously about teacher bias with behavior-specific praise and reprimands for different demographic groups. A behavior-analytic intervention led to praise equity for the students. First, teachers were trained to use self-monitoring and received performance feedback on the overall number of behavior-specific praise and reprimands delivered each session. Next, they received training about the importance of equalizing behavior-specific praise across all students. Finally, performance feedback was altered to include information about the top three students praised and reprimanded and the bottom three students praised and reprimanded. After the final phases of training, children in all demographic categories received equitable rates of praise and reprimands.

Practitioners can collect data about racist behaviors, identify functions, develop an intervention, increase monitoring and reporting of prejudicial behaviors, reinforce alternative behaviors, or punish prejudice through social contingencies. Many potential prejudice reduction procedures fit neatly with traditional behavioral methods, such as reinforcing interracial socializing, altering listener responding, penalizing overt racism, modeling, developing relationships (see Arhin & Thyer, 2004, for a review), counterreasoning (Flannelly & Flannelly, 2000), and behavioral training (Eilers, 2019) or perspective taking (McHugh et al., 2019; Peters & Thompson, 2018). Practitioners can apply these approaches to everyday racial prejudice situations such as bullying, segregation, slurs, health access disparities, and hiring bias. They can also combine applied behavior analysis with ACTr interventions such as value clarification (Cohen et al., 2006; Cohen, Garcia, Purdie-Vaugns, Apfel, & Brzustoski, 2009), mindfulness (Hooper et al., 2010; Lueke & Gibson, 2015, 2016; Proulx et al., 2018), and perspective taking (Lai et al., 2016; Lillis & Hayes, 2007; Todd et al., 2011; Todd et al., 2012).

## Part IV: Call for Collaboration Around Racism

There is room for much more behavior-analytic work targeting racial prejudice. Though it might take time and joint efforts by behavior-analytic, mindfulness, and ACTr researchers, the reduction of racism, bias, and prejudice is a worthy aim. Evidence is growing that derived relational technology, including mindfulness and ACTr interventions, may lead us to ideas for effective interventions (Critchfield et al., 2018; Dixon et al., 2018; Levin et al., 2016; Lillis & Hayes, 2007; Miller, Cruz, & Ala’i-Rosales, 2019; Todd et al., 2011). What more can behavior analysis do to create meaningful, lasting change on a larger scale?

Overall, those in our scientific community should continue to investigate the emerging and promising procedures described thus far. Together, we can research and implement behavioral methods to mitigate prejudiced behaviors. As our profession grows globally (Behavior Analyst Certification Board, 2019), professionals will no doubt witness racism from a new perspective. We hope readers will be inspired to think bigger and expanding to our broader communities. Furthermore, few studies have experimentally analyzed racial bias reduction or the attenuation of its effect on those who experience it.

Behavior analysts can follow many possible next steps. For example, we can do the following:

- Try to recognize and attenuate our personal and professional biased behaviors.
- Collect data to assess prejudicial behaviors in applied settings.
- Study the racial experience of professionals and those we serve in applied behavior analysis.
- Extend and replicate current studies to more diverse populations.
- Compare or combine components of ACTr and applied behavior analysis for prejudice reduction.
- Develop interventions to address the private events involved in racial prejudice.
- Explore strategies for reducing overt prejudiced behaviors in varied settings.
- Create organizational procedures to systematically reduce prejudicial bias.
- Conduct longitudinal studies of intervention outcomes.
- Develop community-wide interventions for reducing prejudice.
- Develop procedures to attenuate the effects of racism on victims.
- Ensure published articles include precise keywords if related to prejudice or racism.^2^

We should also try to improve service delivery to diverse populations that may be affected by racial bias from providers (Brodhead, Quigley, & Wilczynski, 2018; Fong, Ficklin, & Lee, 2017). Unfortunately, recent survey data show that behavior analysts have minimal experience working with diverse cultural minorities and lack the training needed to respond to the multifaceted difficulties these groups face (Beaulieu, Addington, & Almeida, 2018; Conners, Johnson, Duarte, Murriky, & Marks, 2019). It is also likely that behavior analysts have little training in implementing ACTr-related interventions with diverse groups. For example, researchers have conducted mindfulness-based interventions in the broader White community (DeLuca, Kelman, & Waelde, 2018), but a recent call has been made for their adaptation to working with ethnic and racial minorities (Proulx et al., 2018).

The studies reviewed here suggest we can have a positive effect on race and prejudice using the tools and strategies described. The number of ACTr-related studies published by behavior analysts is on the rise (e.g., Brazeau et al., 2017; Castro et al., 2016; Eilers & Hayes, 2015; Enoch & Dixon, 2017; Gould et al., 2018; Hahs et al., 2018; Paliliunas et al., 2018; Rosales, Jowett Hirst, Garcia, & Rehfeldt, 2019), and the topic is in at least one commonly used behavior analysis textbook (Cooper, Heron, & Heward, 2020). However, it is unclear how many behavior analysts are aware of or competent in the use of ACTr-related and mindfulness strategies. We have no data yet on the use of these technologies in more traditional applied behavior-analytic settings but suspect most behavior analysts receive little to no training to integrate these specific strategies into their practice. We are currently collecting related data on this topic.

## Summary

This review presents studies of processes underlying racial bias and prejudice in humans. Behavior analysis has produced little research about direct-acting contingencies that lead to or attenuate racism or bias. Researchers in the field of DRR and ACTr-related procedures have accumulated a small body of research that may help us understand how racist views originate. Additionally, they have evaluated or piloted various individualized interventions to overcome some adverse effects of racial bias.

This article does not present a definitive solution to this complex social problem because the current behavioral research is neither sufficient nor robust. Instead, we are offering behavior analysts a summary of research deficits and potential applications for research and intervention. We encourage readers to be open and curious about bias and racism reduction research across a variety of approaches. We discourage professionals from focusing exclusively on RFT and ACTr or solely traditional behavioral accounts and hope they will join efforts in combining technologies to maximize outcomes. Racism is a devastating community and global issue. Working together, we can better understand and reduce racism and prejudice.

## Literature Search Parameters

### Search 1

PsychInfo database, 1990 to current, select “Peer-reviewed.”

Pub.Exact(“Analysis of Verbal Behavior” OR “The Behavior Analyst” OR “Behavior and Philosophy” OR “Journal of Applied Behavior Analysis” OR “Journal of Organizational Behavior Management” OR “Behavior Modification” OR “Journal of the Experimental Analysis of Behavior” OR “Behavior Analysis: Research and Practice” OR “Behavioural Processes” OR “Behavior and Social Issues” OR “The Behavior Analyst Today” OR “Behavior Analysis in Practice” OR “The Psychological Record” OR “Behavioral Interventions”) AND ti(racism OR racial OR bias OR prejudice OR racism OR stigma OR ethnicity)

### Search 2

PsychInfo database, 1990 to current, select “Peer-reviewed.”

Pub.Exact(“Analysis of Verbal Behavior” OR “The Behavior Analyst” OR “Behavior and Philosophy” OR “Journal of Applied Behavior Analysis” OR “Journal of Organizational Behavior Management” OR “Behavior Modification” OR “Journal of the Experimental Analysis of Behavior” OR “Behavior Analysis: Research and Practice” OR “Behavioural Processes” OR “Behavior and Social Issues” OR “The Behavior Analyst Today” OR “Behavior Analysis in Practice” OR “The Psychological Record” OR “Behavioral Interventions”) AND ti(Black OR White OR Latin)

## Footnotes

1 Please notify the corresponding author if we have missed examples of such studies.

2 *Racism* and *prejudice* are words that may engender a strong emotional reaction. However, less harsh words such as *bias* and *discrimination* already have special technical meanings in applied behavior analysis and research. Thus, using them as keywords may not help researchers correctly classify work so others in the field can locate it.

## References

[CR1] Arhin, A., & Thyer, B. A. (2004). The causes of racial prejudice: A behavior analytic perspective. In J. L. Chin (Ed.), *The psychology of racial prejudice and discrimination*, *Racism in America* (Vol. *I*, pp. 1–19). Westport, CT: Praeger.

[CR2] Barnes-Holmes D, Murphy A, Barnes-Holmes Y, Stewart I (2010). The implicit relational assessment procedure: Exploring the impact of private versus public contexts and the response latency criterion on pro-White and anti-Black stereotyping among White Irish individuals. The Psychological Record.

[CR3] Beaulieu L, Addington J, Almeida D (2018). Behavior analysts’ training and practices regarding cultural diversity: The case for culturally competent care. Behavior Analysis in Practice.

[CR4] Behavior Analyst Certification Board. (2019). *BCBA examination pass rates for verified course sequences 2014–2018*. Retrieved from https://www.bacb.com/wp-content/uploads/BCBA-Pass-Rates-Alpha_190813.pdf

[CR5] Brazeau K, Rehfeldt RA, Mazo A, Smalley S, Krus S, Henson L (2017). On the efficacy of mindfulness, defusion, and behavioral skills training on job interviewing skills in dually-diagnosed adults with developmental disorders. Journal of Contextual Behavioral Science.

[CR6] Briggs HE, Paulson RI, Mattaini MA, Thyer BA (1996). Racism. *Finding solutions to social problems: Behavioral strategies for change*.

[CR7] Brodhead MT, Quigley SP, Wilczynski SM (2018). A call for discussion about scope of competence in behavior analysis. Behavior Analysis in Practice.

[CR8] Castro M, Rehfeldt RA, Root WB (2016). On the role of values clarification and committed actions in enhancing the engagement of direct care workers with clients with severe developmental disorders. Journal of Contextual Behavioral Science.

[CR9] Chancey C, Weihl C, Root WB, Rehfeldt RA, McCauley D, Takeguchi K, Pritchard J (2018). The impact of mindfulness skills on interactions between direct care staff and adults with developmental disabilities. Journal of Contextual Behavioral Science.

[CR10] Cohen GL, Garcia J, Apfel N, Master A (2006). Reducing the racial achievement gap: A social-psychological intervention. Science.

[CR11] Cohen GL, Garcia J, Purdie-Vaugns V, Apfel N, Brzustoski P (2009). Recursive processes in self-affirmation: Intervening to close the minority achievement gap. Science.

[CR12] Conners B, Johnson A, Duarte J, Murriky R, Marks K (2019). Future directions of training and fieldwork in diversity issues in applied behavior analysis. Behavior Analysis in Practice.

[CR13] Cooper JO, Heron TE, Heward WL (2020). *Applied behavior analysis*.

[CR14] Corti C, Pergolizzi F, Vanzin L, Cargasacchi G, Villa L, Pozzi M, Molteni M (2018). Acceptance and commitment therapy-oriented parent-training for parents of children with autism. Journal of Child and Family Studies.

[CR15] Critchfield TS, Barnes-Holmes D, Dougher MJ (2018). What Sidman did: Historical and contemporary significance of research on derived stimulus relations. Perspectives on Behavioral Science.

[CR16] de Carvalho MP, de Rose JC (2014). Understanding racial attitudes through the stimulus equivalence paradigm. The Psychological Record.

[CR17] De Houwer J (2019). Implicit bias is behavior: A functional-cognitive perspective on implicit bias. Perspectives on Psychological Science.

[CR18] DeLuca SM, Kelman AR, Waelde LC (2018). A systematic review of ethnoracial representation and cultural adaptation of mindfulness- and meditation-based interventions. Psychological Studies.

[CR19] Dixon MR, Belisle J, Rehfeldt RA, Root WB (2018). Why we are still not acting to save the world: The upward challenge of a post-Skinnerian behavior science. Perspectives on Behavior Science.

[CR20] Dixon MR, Branon A, Nastally BL, Mui N (2009). Examining prejudice towards Middle Eastern persons via a transformation of stimulus functions. The Behavior Analyst Today.

[CR21] Dixon MR, Lemke M (2007). Reducing prejudice towards Middle Eastern persons as terrorists. European Journal of Behavior Analysis.

[CR22] Dixon MR, Paliliunas D, Belisle J, Speelman RC, Gunnarsson KF, Shaffer JL (2019). The effect of brief mindfulness training on momentary impulsivity. Journal of Contextual Behavioral Science.

[CR23] Dixon MR, Rehfeldt RA, Zlomke KR, Robinson A (2006). Exploring the development and dismantling of equivalence classes involving terrorist stimuli. The Psychological Record.

[CR24] Dixon MR, Wilson AN, Habib R (2016). Neurological evidence of acceptance and commitment therapy effectiveness in college-age gamblers. Journal of Contextual Behavioral Science.

[CR25] Dixon MR, Zlomke KM, Rehfeldt RA (2006). Restoring Americans’ nonequivalent frames of terror: An application of relational frame theory. The Behavior Analyst Today.

[CR26] Donnchadha SÓ (2018). Stress in caregivers of individuals with intellectual or developmental disabilities: A systematic review of mindfulness-based interventions. Journal of Applied Research in Intellectual Disabilities.

[CR27] Drake CE, Kramer S, Sain T, Swiatek R, Kohn K, Murphy M (2015). Exploring the reliability and convergent validity of implicit racial evaluations. Behavior and Social Issues.

[CR28] Edwards DJ, McEnteggart C, Barnes-Holmes Y, Lowe R, Evans N, Vilardaga R (2017). The impact of mindfulness and perspective-taking on implicit associations toward the elderly: A relational frame theory account. Mindfulness.

[CR29] Eilers HJ (2019). The utility of a function-based approach to intimate partner violence and gender bias in family courts. Behavior Analysis in Practice.

[CR30] Eilers HJ, Hayes SC (2015). Exposure and response prevention therapy with cognitive defusion exercises to reduce repetitive and restrictive behaviors displayed by children with autism spectrum disorder. Research in Autism Spectrum Disorders.

[CR31] Enoch MR, Dixon MR (2017). The use of a child-based acceptance and commitment therapy curriculum to increase attention. Child & Family Behavior Therapy.

[CR32] Fix R, Fix S (2013). The effects of mindfulness-based treatments for aggression: A critical review. Aggression and Violent Behavior.

[CR33] Flannelly LT, Flannelly KJ (2000). Reducing people’s judgment bias about their level of knowledge. The Psychological Record.

[CR34] Fletcher L, Hayes SC (2005). Relational frame theory, acceptance and commitment therapy, and a functional analytic definition of mindfulness. Journal of Rational-Emotive and Cognitive-Behavior Therapy.

[CR35] Fong EH, Ficklin S, Lee HY (2017). Increasing cultural understanding and diversity in applied behavior analysis. Behavior Analysis: Research and Practice.

[CR36] Galinsky AD, Moskowitz GB (2000). Perspective-taking: Decreasing stereotype expression, stereotype accessibility, and in-group favoritism. Journal of Personality and Social Psychology.

[CR37] Gifford EV, Hayes SC, O’Donohue W, Kitchener R (1999). Functional contextualism: A pragmatic philosophy for behavioral science. *Handbook of behaviorism*.

[CR38] Godfrey K, Gallo L, Afari N (2015). Mindfulness-based interventions for binge eating: A systematic review and meta-analysis. Journal of Behavioral Medicine.

[CR39] Golijani-Moghaddam N, Hart A, Dawson DL (2013). The implicit relational assessment procedure: Emerging reliability and validity data. Journal of Contextual Behavioral Science.

[CR40] Gould ER, Tarbox J, Coyne L (2018). Evaluating the effects of acceptance and commitment training on the overt behavior of parents of children with autism. Journal of Contextual Behavioral Science.

[CR41] Guerin B (2003). Combating prejudice and racism: New interventions from a functional analysis of racist language. Journal of Community & Applied Social Psychology.

[CR42] Guerin B (2005). Combating everyday racial discrimination without assuming “racists” or “racism”: New intervention ideas from a contextual analysis. Behavior and Social Issues.

[CR43] Hahs AD, Dixon MR, Paliliunas D (2018). Randomized controlled trial of a brief acceptance and commitment training for parents of individuals diagnosed with autism spectrum disorders. Journal of Contextual Behavioral Science.

[CR44] Hauserman N, Walen SR, Behling M (1973). Reinforced racial integration in the first grade: A study in generalization. Journal of Applied Behavior Analysis.

[CR45] Hayes SC, Levin ME, Plumb-Vilardaga J, Villatte JL, Pistorello J (2013). Acceptance and commitment therapy and contextual behavioral science: Examining the progress of a distinctive model of behavioral and cognitive therapy. Behavior Therapy.

[CR46] Hoffmann AN, Contreras BP, Clay CJ, Twohig MP (2016). Acceptance and commitment therapy for individuals with disabilities: A behavior analytic strategy for addressing private events in challenging behavior. Behavior Analysis in Practice.

[CR47] Hooper N, Villatte M, Neofotistou E, McHugh L (2010). The effects of mindfulness versus thought suppression on implicit and explicit measures of experiential avoidance. International Journal of Behavioral Consultation and Therapy.

[CR48] Katterman S, Kleinman B, Hood M, Nackers L, Corsica J (2014). Mindfulness meditation as an intervention for binge eating, emotional eating, and weight loss: A systematic review. Eating Behaviors.

[CR49] Khoury B, Lecomte T, Gaudiano B, Paquin K (2013). Mindfulness interventions for psychosis: A meta-analysis. Schizophrenia Research.

[CR50] Kishita N, Muto T, Ohtsuki T, Barnes-Holmes D (2014). Measuring the effect of cognitive defusion using the implicit relational assessment procedure: An experimental analysis with a highly socially anxious sample. Journal of Contextual Behavioral Science.

[CR51] Klingbeil D, Fischer A, Renshaw T, Bloomfield B, Polakoff B, Willenbrink J, Chan K (2017). Effects of mindfulness-based interventions on disruptive behavior: A meta-analysis of single-case research. Psychology in the Schools.

[CR52] Knochel, A. E., Blair, K. C., Kincaid, D., & Randazzo, A. (2019). *Promoting equity in teachers’ use of behavior-specific praise with self-monitoring and performance feedback.* Manuscript submitted for publication.

[CR53] Kohlenberg BS, Hayes SC, Hayes LJ (1991). The transfer of contextual control over equivalence classes through equivalence classes: A possible model of social stereotyping. Journal of the Experimental Analysis of Behavior.

[CR54] Krafft J, Ferrell J, Levin ME, Twohig MP (2018). Psychological inflexibility and stigma: A meta-analytic review. Journal of Contextual Behavioral Science.

[CR55] Lai CK, Skinner AL, Cooley E, Murrar S, Brauer M, Devos T (2016). Reducing implicit racial preferences: II. Intervention effectiveness across time. Journal of Experimental Psychology: General.

[CR56] Leeming E, Hayes SC (2016). Parents are people too: The importance of parental psychological flexibility. Clinical Psychology: Science and Practice.

[CR57] Levin ME, Luoma JB, Vilardaga R, Lillis J, Nobles R, Hayes SC (2016). Examining the role of psychological inflexibility, perspective taking, and empathic concern in generalized prejudice. Journal of Applied Social Psychology.

[CR58] Lillis J, Hayes SC (2007). Applying acceptance, mindfulness, and values to the reduction of prejudice: A pilot study. Behavior Modification.

[CR59] Lueke A, Gibson B (2015). Mindfulness meditation reduces implicit age and race bias: The role of reduced automaticity of responding. Social Psychological and Personality Science.

[CR60] Lueke A, Gibson B (2016). Brief mindfulness meditation reduces discrimination. Psychology of Consciousness: Theory, Research, and Practice.

[CR61] Lunsky Y, Fung K, Lake J, Steel L, Bryce K (2018). Evaluation of acceptance and commitment therapy (ACT) for mothers of children and youth with autism spectrum disorder. Mindfulness.

[CR62] McGlinchey, A., & Keenan, M. (1997). Stimulus equivalence and social categorization in Northern Ireland. *Behavior and Social Issues, 7*(2), 113–128. 10.5210/bsi.v7i2.310.

[CR63] McHugh, L., Stewart, I., & Almada, P. (2019). *A contextual behavioral guide to the self: Theory and practice*. New Harbinger Publications.

[CR64] Miller, K. L., Cruz, A. R., & Ala’i-Rosales, S. (2019). Inherent tensions and possibilities: Behavior analysis and cultural responsiveness. *Behavior and Social Issues 28*(1), 16-36.

[CR65] Mizael TM, de Almeida JH, Silveira CC, de Rose JC (2016). Changing racial bias by transfer of functions in equivalence classes. The Psychological Record.

[CR66] Paliliunas D, Belisle J, Dixon MR (2018). A randomized control trial to evaluate the use of acceptance and commitment therapy (ACT) to increase academic performance and psychological flexibility in graduate students. Behavior Analysis in Practice.

[CR67] Parker SK, Atkins PW, Axtell CM (2008). Building better workplaces through individual perspective taking: A fresh look at a fundamental human process. International Review of Industrial and Organizational Psychology.

[CR68] Pennefather J, Hieneman M, Raulston TJ, Caraway N (2018). Evaluation of an online training program to improve family routines, parental well-being, and the behavior of children with autism. Research in Autism Spectrum Disorders.

[CR69] Peters LC, Thompson RH (2018). How teaching perspective taking to individuals with autism spectrum disorders affects social skills: Findings from research and suggestions for practitioners. Behavior Analysis in Practice.

[CR70] Power PM, Harte C, Barnes-Holmes D, Barnes-Holmes Y (2017). Exploring racial bias in a country with a recent history of immigration of Black Africans. The Psychological Record.

[CR71] Prejudice. (2019). In *Oxford English Dictionary online*. Retrieved from https://en.oxforddictionaries.com/definition/prejudice

[CR72] Proulx J, Croff R, Oken B, Aldwin CM, Fleming C, Bergen-Cico D, Noorani M (2018). Considerations for research and development of culturally relevant mindfulness interventions in American minority communities. Mindfulness.

[CR73] Racism. (2019a). In Merriam-Webster.com. Retrieved from https:// www.merriam-webster.com/dictionary/racism

[CR74] Racism. (2019b). In *Oxford English Dictionary online.* Retrieved from https://en.oxforddictionaries.com/definition/racism

[CR75] Ritzert TR, Forsyth JP, Berghoff CR, Barnes-Holmes D, Nicholson E (2015). The impact of a cognitive defusion intervention on behavioral and psychological flexibility: An experimental evaluation in a spider fearful non-clinical sample. Journal of Contextual Behavioral Science.

[CR76] Roche B, Barnes-Holmes Y, Barnes-Holmes D, Stewart I, O’Hora D (2002). Relational frame theory: A new paradigm for the analysis of social behavior. The Behavior Analyst.

[CR77] Rosales R, Jowett Hirst ES, Garcia Y, Rehfeldt RA (2019). Autism beyond early intensive behavioral intervention. Advances in Neurodevelopmental Disorders.

[CR78] Shonin E, Van Gordon W, Slade K, Griffiths MD (2013). Mindfulness and other Buddhist-derived interventions in correctional settings: A systematic review. Aggression and Violent Behavior.

[CR79] Skinner, A. L., Olson, K. R., & Meltzoff, A. N. (2019). Acquiring group bias: Observing other people’s nonverbal signals can create social group biases. *Journal of Personality and Social Psychology. Advance online publication.*10.1037/pspi0000218. 10.1037/pspi0000218 PMC8276637 31524429

[CR80] Stewart I, McElwee J, Ming S (2013). Language generativity, response generalization, and derived relational responding. The Analysis of Verbal Behavior.

[CR81] Szabo, T. G. (2019). Acceptance and commitment training for reducing inflexible behaviors in children with autism. *Journal of Contextual Behavioral Science. Advance online publication.*10.1016/j.jcbs.2019.03.001.

[CR82] Todd AR, Bodenhausen GV, Galinsky AD (2012). Perspective taking combats the denial of intergroup discrimination. Journal of Experimental Social Psychology.

[CR83] Todd AR, Bodenhausen GV, Richeson JA, Galinsky AD (2011). Perspective taking combats automatic expressions of racial bias. Journal of Personality and Social Psychology.

[CR84] Watt A, Keenan M, Barnes D, Cairns E (1991). Social categorization and stimulus equivalence. The Psychological Record.

[CR85] Wolfgang, H., & Catagnus, R. M. (2019)*. Is mindfulness behavior analytic? A systematic literature review.* Manuscript in preparation.

[CR86] Zettle RD (2005). The evolution of a contextual approach to therapy: From comprehensive distancing to ACT. International Journal of Behavioral Consultation and Therapy.

